# Subcellular forms and biochemical events triggered in human cells by HCV polyprotein expression from a viral vector

**DOI:** 10.1186/1743-422X-5-102

**Published:** 2008-09-15

**Authors:** Andrée M Vandermeeren, Carmen Elena Gómez, Cristina Patiño, Elena Domingo-Gil, Susana Guerra, Jose Manuel González, Mariano Esteban

**Affiliations:** 1 Department of Molecular and Cellular Biology, Centro Nacional de Biotecnología, CSIC, Campus Universidad Autónoma, E-28049, Madrid, Spain; 2 Electron Microscopy Service, Centro Nacional de Biotecnología, CSIC, Campus Universidad Autónoma, E-28049, Madrid, Spain

## Abstract

To identify the subcellular forms and biochemical events induced in human cells after HCV polyprotein expression, we have used a robust cell culture system based on vaccinia virus (VACV) that efficiently expresses in infected cells the structural and nonstructural proteins of HCV from genotype 1b (VT7-HCV7.9). As determined by confocal microscopy, HCV proteins expressed from VT7-HCV7.9 localize largely in a globular-like distribution pattern in the cytoplasm, with some proteins co-localizing with the endoplasmic reticulum (ER) and mitochondria. As examined by electron microscopy, HCV proteins induced formation of large electron-dense cytoplasmic structures derived from the ER and containing HCV proteins. In the course of HCV protein production, there is disruption of the Golgi apparatus, loss of spatial organization of the ER, appearance of some "virus-like" structures and swelling of mitochondria. Biochemical analysis demonstrate that HCV proteins bring about the activation of initiator and effector caspases followed by severe apoptosis and mitochondria dysfunction, hallmarks of HCV cell injury. Microarray analysis revealed that HCV polyprotein expression modulated transcription of genes associated with lipid metabolism, oxidative stress, apoptosis, and cellular proliferation. Our findings demonstrate the uniqueness of the VT7-HCV7.9 system to characterize morphological and biochemical events related to HCV pathogenesis.

## Background

Hepatitis C virus (HCV) infection is a major cause of chronic hepatitis, liver cirrhosis and hepatocellular carcinoma [1]. With over 170 million people chronically infected with HCV worldwide, this disease has emerged as a serious global health problem.

The HCV virus is the sole member of the genus hepacivirus which belongs to the *Flaviviridae *family, represented by six major genotypes. The viral genome is a positive polarity single-stranded RNA molecule of approximately 9.5 kb in length that has a unique open-reading frame, coding for a single polyprotein. The length of the polyprotein-encoding region varies according to the isolate and genotype of the virus from 3,008 to 3,037 amino acids. After virus entry and uncoating, the viral genome serves as template for the translation of the single polyprotein which is processed by cellular and viral proteases to yield the mature structural (Core-E1-E2-p7) and nonstructural proteins (NS2-NS3-NS4A-NS4B-N5A-NS5B) [2,3].

Despite the identification of HCV as the most common etiologic agent of posttransfusion and sporadic non-A, non-B hepatitis, its replication cycle and pathogenesis are incompletely understood. Improvement has been made using heterologous expression systems, functional full-length cDNA clones, and subgenomic replicons [4-6]. The recent establishment of a cell culture system for HCV propagation is a major progress to analyse the complete viral life cycle and HCV virus-host interactions [7-9].

The impact of HCV polyprotein expression in human cells has been hampered by limitations of different cell systems to express the entire HCV polyprotein in high yields and in all cells. Vaccinia virus (VACV), a prototype member of the poxvirus family, has proven to be a useful vector for faithful expression of many proteins in cells [10,11]. We have previously described a novel poxvirus-based delivery system that is inducible and expresses the structural and nonstructural (except C-terminal part of NS5B) proteins of HCV ORF from genotype 1b [12]. In this model, we observed that HCV proteins control cellular translation through eIF-2α-S51 phosphorylation, with involvement of the double-stranded RNA-dependent protein kinase PKR. Moreover, in VT7-HCV7.9 infected cells HCV proteins bring about an apoptotic response through the activation of the RNase L pathway [12].

As it has been considered that the viral cytopathic effect might be involved in the liver-cell injuries [1,2,13], here we have analyzed in detail the subcellular forms and biochemical changes occurring in human cells (HeLa and hepatic HepG2) following expression of the HCV polyprotein from VACV recombinant. We found that the production of HCV proteins in the host cell from 4 to 48 h induced severe cellular damage with modifications in cell organelles, formation of large cytoplasmic membrane structures and activation of death pathways, hallmarks of HCV cell injury. In addition, we analyzed by microarray technology the gene expression profile of HeLa cells infected with VT7-HCV7.9 recombinant and identified genes that were differentially regulated by HCV proteins and are related with HCV pathogenesis. The morphological and biochemical changes triggered in human cells by HCV polyprotein expression highlight the use of the poxvirus-based system as a suitable model in the study of the biology of HCV infection and morphogenesis, host-cell interactions and drug-treatment.

## Results

### HCV proteins induced disruption of the Golgi apparatus and co-localized with ER and mitochondria markers

We have previously described that the DNA fragment of HCV ORF from genotype 1b included in the VT7-HCV_7.9 _recombinant is efficiently transcribed and translated upon induction with IPTG into a viral polyprotein precursor that is correctly processed into mature structural and nonstructural (except the C-terminal part of NS5B) HCV proteins [12].

**Figure 1:**
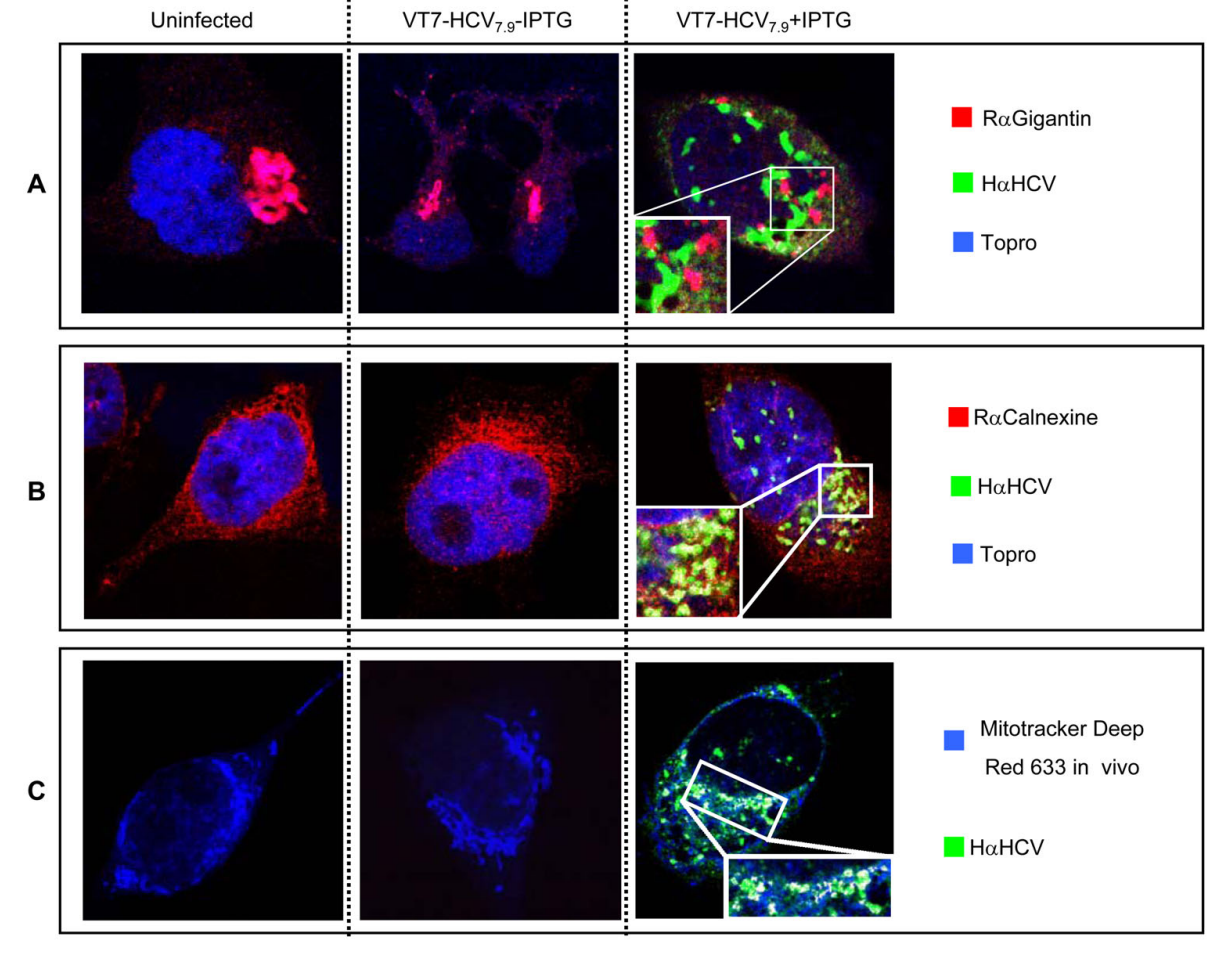
**Compartmentalization of HCV proteins produced in HeLa cells infected with VT7-HCV_7.9_**. Subconfluent HeLa cells uninfected or infected at 5 PFU/cell with the recombinant VT7-HCV7.9 in the presence (+) or absence (-) of the inducer IPTG, were fixed at 24 h p.i, labelled with the corresponding primary antibody followed by the appropriate fluorescent secondary antibody and visualized by confocal microscopy. The antibodies or reagents used were Hα HCV to detect HCV proteins; Topro-3 to detect DNA; Rα Giantin to detect the Golgi complex **(A)**; Rα Calnexine to detect the endoplasmatic reticulum **(B) **and Mitotracker Deep Red 633 to detect mitochondria **(C)**. The co-localization is shown with a higher resolution in the white square.

To identify the cytoplasmic compartment(s) in which viral HCV proteins accumulated during infection of HeLa cells with VT7-HCV_7.9_, we performed immunofluorescence analysis using serum from an HCV-infected patient to recognize HCV proteins and antibodies specific for the Golgi apparatus (anti-gigantin), the endoplasmic reticulum (anti-calnexin) or the mitochondria (mitotracher) (Fig. 1). Inducible expression of HCV proteins caused severe disruption of the Golgi apparatus as revealed by labelling this organelle with a specific antibody (Fig. 1A). This effect was not observed in cells infected with VT7-HCV_7.9 _in the absence of IPTG. There is no co-localization of HVC proteins with the disrupted Golgi, whereas in the labelling of the endoplasmic reticulum, a clear co-localization between HCV proteins expressed from VT7-HCV_7.9 _and ER proteins was observed (Fig. 1B). With an *in vivo *mitochondrial marker (Fig. 1C), we detected partial co-localization between HCV proteins expressed from VT7-HCV_7.9 _and mitochondria organelles. Moreover, the mitochondria appeared more rounded in cells infected with VT7-HCV_7.9 _+ IPTG, in comparison with uninfected cells or with cells infected in the absence of IPTG.

### HCV polyprotein expression in human HeLa and HepG2 cells induces severe morphological alterations and production of electron dense structures in the cytoplasm surrounded by membranes

**Figure 2:**
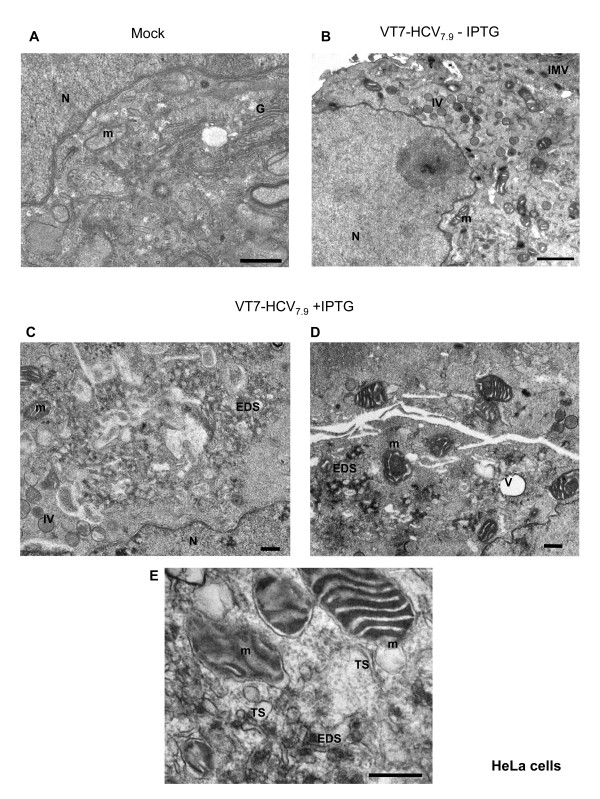
**Alterations in the architecture of HeLa cells following expression of HCV proteins from VT7-HCV_7.9 _seen by electron microscopy**. HeLa cells uninfected or infected with the recombinant VT7-HCV7.9 in the presence or absence of the inducer IPTG, were chemically fixed at 16 h p.i and then processed for conventional embedding in an epoxy resin as described under Materials and Methods.**A: **Cellular architecture of uninfected HeLa cells. **B: **A general view of a cell infected with VT7-HCV7.9 in the absence of IPTG, showing the VACV forms IVs and IMVs. **C and D: **A general view of cells infected with VT7-HCV7.9 in the presence of IPTG, showing few IVs, large EDS, swollen mitochondria and vacuoles. **E: **High magnification of infected VT7-HCV7.9 cells in the presence of IPTG showing EDS with membranes, TS and swollen mitochondria with a protruding membrane. Note: Nucleus (N), mitochondria (m), Golgi apparatus (G), immature virus (IV), intracellular mature virus (IMV), tubular structures (TS), electron dense structures in membranous webs (EDS). Bar: 500 nm.

To gain more detail information on the subcellular changes induced by HCV polyprotein expression, we performed transmission electron microscopy (EM) analysis. HeLa cells were infected with VT7-HCV_7.9 _in the presence or absence of IPTG, and at 16 h p.i, infected and uninfected cells were collected and ultrathin sections visualized by EM at low and high magnification. While in cells infected with VT7-HCV_7.9_, in the absence of IPTG there are high number of immature (IVs) and intracellular mature (IMVs) forms of VACV virus, in cells infected with VT7-HCV_7.9 _in the presence of IPTG fewer IVs and IMVs were observed, corroborating our previous finding that the expression of HCV proteins blocked VACV morphogenesis [12]. In addition, several morphological alterations were detected in cells expressing the HCV polyprotein when compared with uninfected cells (Fig. 2A) or with cells only expressing VACV proteins (Fig. 2B). The first alteration seen was the loss of spatial organization of the ER, with vesicles embedded in a membrane matrix of circular or tightly undulating membranes, forming electron dense structures indicated as EDS (Fig. 2C and 2D). These cytoplasmic structures resemble those structures called "membraneous webs" that have been visualized in human hepatoma Huh7 cells expressing a subgenomic HCV replicon [5,14,15]. Other alterations observed were the presence of vacuoles (indicated as V) often surrounding the compact structures, as well as the presence of swollen mitochondria (indicated as m) (Fig. 2D and 2E). Higher magnification of the electron dense structures in cells expressing the HCV polyprotein revealed membranes and tubular structures (indicated as TS) as part of the EDS (Fig. 2E).

**Figure 3:**
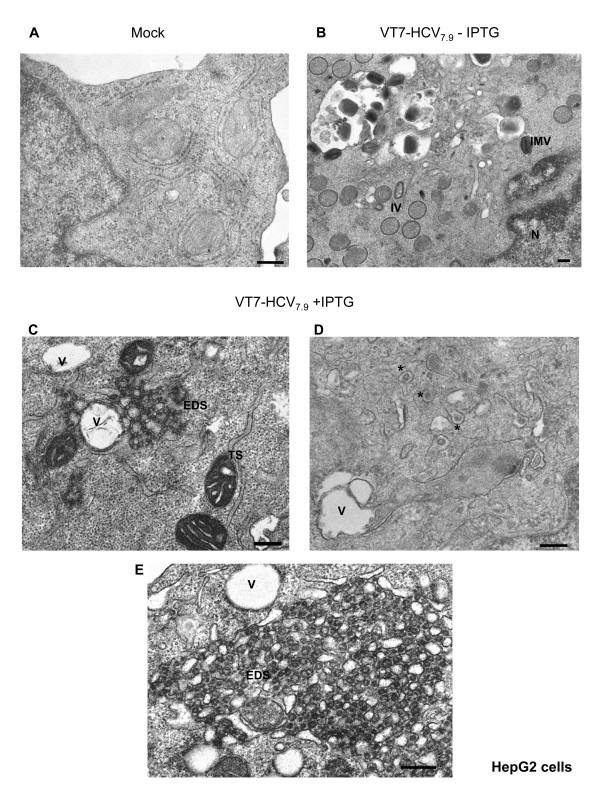
**Hepatocyte cell alterations following infection of HepG2 with VT7-HCV7.9**. HepG2 cells uninfected or infected with the recombinant VT7-HCV7.9 in the presence or absence of the inducer IPTG were chemically fixed at 16 h p.i and then processed for conventional embedding in an epoxy resin.**A: **Cellular architecture of uninfected HepG2 cells. **B**: A general view of a cell infected with VT7-HCV7.9 in the absence of IPTG, showing the VACV forms IVs and IMVs.**C, D and E: **A general view of a cell infected with VT7-HCV7.9 in the presence of IPTG, showing large EDS surrounded by vacuoles and the presence of "virus-like particles" surrounded with membranes (*). Note: Vacuole (V) and electron dense structures in membranous webs (EDS). Bar: 200 nm.

Since hepatocytes are the main targets of HCV virus, we next analyzed if expression of HCV polyprotein from the VT7-HCV_7.9 _infected cells also affected the ultra-structure of hepatic cells. Thus, monolayers of a human hepatoblast cell line (HepG2) were infected with VT7-HCV_7.9 _under the same conditions as for HeLa cells and processed at 16 h p.i for EM analysis. In this cell line, similarly as in HeLa cells, the inducible expression of HCV proteins blocks VACV morphogenesis and induces the same alterations described above. In contrast to uninfected (Fig 3A) and infected hepG2 cells in absence of IPTG (Fig. 3B), in cells expressing HCV proteins we distinguish EDS in membranous webs (Fig. 3C and 3E), formation of large vacuoles (Fig. 3C and 3D), and also identified "virus like-particles" structures surrounded by membranes and dispersed in several areas of the cell cytoplasm (Fig. 3D).

### Immunogold electron microscopy revealed that HCV proteins are part of the electron dense and membranous structures

**Figure 4:**
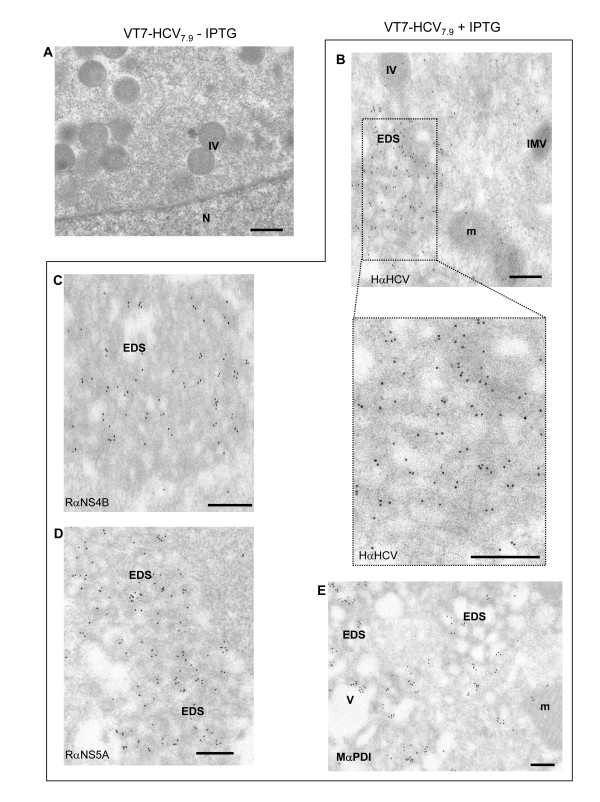
**Immunogold electron microscopy analysis of the localization of HCV proteins in VT7-HCV_7.9 _infected HeLa cells**. HeLa cells infected with VT7-HCV7.9 in the presence or absence of IPTG were chemically fixed, quickly frozen in liquid propane and then processed at low temperature in Lowicryl K4M resin. Immunogold labelling was performed with different antibodies. **A**: Cells infected with VT7-HCV7.9 in the absence of IPTG reacted with a serum from an HCV-infected patient.**B**: Cells infected with VT7-HCV7.9 in the presence of IPTG reacted with a serum from an HCV-infected patient **C**: Cells infected with VT7-HCV7.9 in the presence of IPTG reacted with a rabbit polyclonal anti-NS4B. **D: **Cells infected with VT7-HCV7.9 in the presence of IPTG reacted with a rabbit polyclonal anti-NS5A. **E**: Cells infected with VT7-HCV7.9 in the presence of IPTG reacted with a mouse monoclonal antibody anti-PDI. Note: Electron dense structures in membranous webs (EDS); mitochondria (m), immature virus (IV), intracellular mature virus (IMV), nucleus (N) and Vacuole (V). Bar: 250 nm.

To assure that the electron dense structures appearing in the cytoplasm of infected cells are the result of HCV polyprotein expression, we performed immunogold electron microscopy analysis with antibodies against HCV structural and nonstructural proteins (Fig. 4). Thus, HeLa cells were infected with VT7-HCV_7.9 _in the presence or absence of IPTG and at 16 h p.i, infected and uninfected cells were processed for immunogold labelling on ultrathin sections. Due to the fixation and embedding procedures used in immunostaining, the cell structures are less visible than by embedding in an epoxy resin. While in cells infected with VT7-HCV_7.9 _in absence of IPTG there was no specific labelling detected with the serum from an HCV-infected patient (Fig. 4A), in contrast, in antibody-reacted cells expressing HCV proteins most gold particles were concentrated into electron dense and membranous structures (Fig. 4B). Discrete labelling was observed in other parts of the cell cytoplasm. The localization of some of the nonstructural HCV proteins was determined using rabbit polyclonal antibodies against NS4B or NS5A proteins. The membranous and electron dense structures were also specifically recognized by antibodies against NS4B (Fig. 4C) and NS5A (Fig. 4D), indicating that both proteins are part of electron dense membrane-associated cytoplasmic complexes.

Since by confocal microscopy we observed co-localization between ER and HCV proteins in cells infected with VT7-HCV_7.9 _in the presence of IPTG (see Fig. 1B), we performed immunogold labelling using a specific ER marker (mouse anti-PDI). As seen in Fig. 4E, strong labelling of ER was found in the membranous webs. These results suggest that the membranous webs are ER-derived structures. As the staining pattern corresponds to that obtained with the NS4B or NS5A proteins, the immunogold electron microscopy indicates that the ER is a site where these proteins are localized.

### HCV polyprotein expression results in mitochondrial dysfunction, as revealed by release of cytochrome c, loss of membrane potential and generation of reactive oxygen species (ROS)

The detection by confocal microscopy of the presence of HCV proteins in the mitochondria (see Fig. 1C) suggests that HCV may regulate the mitochondria homeostasis. To confirm that, we evaluated different parameters such as, release of proapototic proteins including cytochrome c, loss of mitochondrial membrane potential (ΔΨm) and production of reactive oxygen species (ROS), all hallmarks of mitochondrial dysfunction.

**Figure 5:**
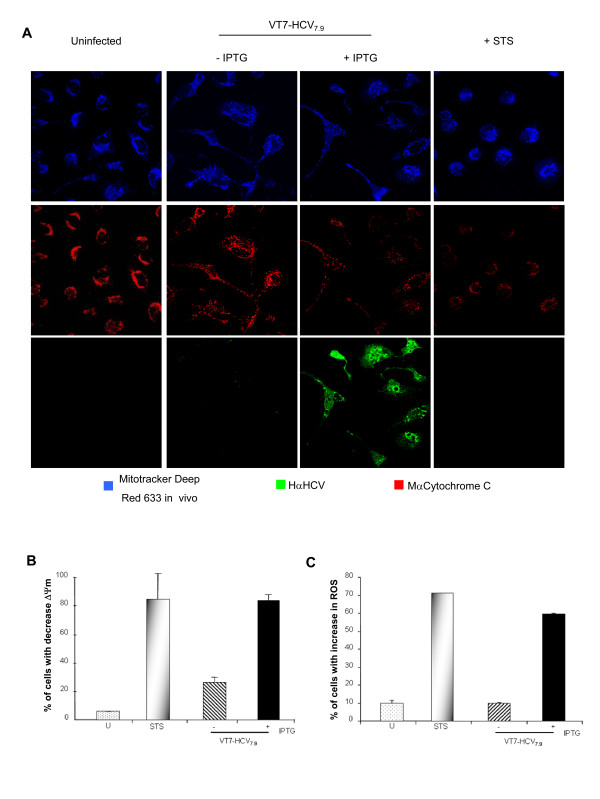
**HCV polyprotein expression induced dysfunction of the mitochondria. A: **HeLa cells uninfected or infected at 5 PFU/cell with the recombinant VT7-HCV7.9 in the presence or absence of IPTG were labelled *in vivo *at 24 h p.i with Mitotracker deep red (blue) to detect the mitochondria, with an anti-cytochrome c (red) antibody and with the serum from an HCV-infected patient to detect HCV proteins. Cells treated with staurosporine at 0.5 μM for 16 h were used as positive control. **B: **HeLa cells were either infected at 5 PFU/cell with the recombinant VT7-HCV7.9 in the presence or absence of IPTG, or treated with staurosporine at 0.5 μM for 16 h. At 48 h p.i, the mitochrondrial membrane potential (ΔΨm) was determined quantifying TMRE fluorescence. **C**: HeLa cells were either infected at 5 PFU/cell with the recombinant VT7-HCV7.9 in the absence or presence of IPTG or treated with staurosporine at 0.5 μM for 16 h. At 48 h p.i, the uninfected and infected cells were stained with dihydroethidium (2-HE) and subjected to flow cytometry. Note: STS: staurosporine.

To determine whether HCV polyprotein expression from the VACV recombinant activates cytochrome c release, HeLa cells were infected with VT7-HCV_7.9 _in the presence or absence of IPTG, or treated with staurosporine (as a positive control). The cytochrome c release was detected by confocal microscopy. As shown in Fig. 5A, the cytochrome c remained confined to the mitochondria in both uninfected cells and VT7-HCV_7.9_infected cells in the absence of IPTG. However, in cells infected with VT7-HCV_7.9 _in the presence of IPTG, there is a diffuse cytosolic pattern of cytochrome c staining, similarly as in cells treated with staurosporine, indicating that cytochrome c was released from the mitochondria.

Next we determine if HCV polyprotein expression affected the mitochondria membrane potential (ΔΨm). HeLa cells were infected either with VT7-HCV_7.9 _in the presence or absence of IPTG, or treated with staurosporine. At 48 h p.i, cells were stained *in vivo *with a fluorescent mitochondrion-specific dye, TMRE [16,17], and analysed by flow cytometry. The loss of the ΔΨm was assessed by the ability of the mitochondria to take up TMRE. As shown in Fig. 5B, FACS analysis demonstrated a higher proportion of cells with decreased ΔΨm (ΔΨm Low) in HCV polyprotein expressing cells and in staurosporine treated cells, in contrast with cells infected with the VT7-HCV_7.9 _in the absence of IPTG or in uninfected cells. These results indicate the ability of the HCV proteins to disrupt the mitochondria membrane potential in HeLa cells.

As mitochondrial dysfunction is also characterized by the generation of reactive oxygen species (ROS) [18], we investigated whether HCV polyprotein expression triggered the generation of ROS. HeLa cells were infected with VT7-HCV_7.9 _in the presence or absence of IPTG and at 48 h p.i, cells were stained with dihydroethidium (2-HE) and subjected to flow cytometry [19]. As shown in Fig. 5C, there is clearly production of ROS, as revealed by an increase in ethidium staining of DNA in HeLa cells infected with VT7-HCV_7.9 _in the presence of IPTG. In contrast, ROS production was significantly lower (about 10%) in both uninfected cells and VT7-HCV_7.9 _infected cells in the absence of IPTG.

The above results demonstrate that HCV proteins induce mitochondrial dysfunction evidenced by the release of cytochrome c, mitochondrial membrane depolarization and generation of ROS.

### Expression of HCV proteins induces apoptosis through activation of initiator and effector caspases

It has been reported in hepatic cells that expression of structural and nonstructural proteins from HCV cDNA [20] or from full-length RNA [21], can lead to apoptotic cell death, which could be an important event in the pathogenesis of chronic HCV infection in humans. We have previously shown by an ELISA-based assay that the inducible expression of HCV proteins from VT7-HCV_7.9 _triggers apoptosis [12]. In view of the severe cellular damage caused by polyprotein expression in VT7-HCV_7.9 _infected cells, we wished to extend our previous observation by characterizing the apoptotic pathways in this virus-cell system. We first performed a qualitative estimation of apoptosis in HeLa cells infected with VT7-HCV_7.9 _in the presence or absence of IPTG. By phase contrast microscopy and DNA staining analysis we observed that cells expressing HCV polyprotein developed at 24 h p.i characteristic morphological changes of apoptosis, as defined by cell shrinkage, granulated appearance, membrane bledding and chromatin condensation (not shown). Such morphological changes were not observed in cells infected with VT7-HCV_7.9 _in the absence of the inducer.

**Figure 6:**
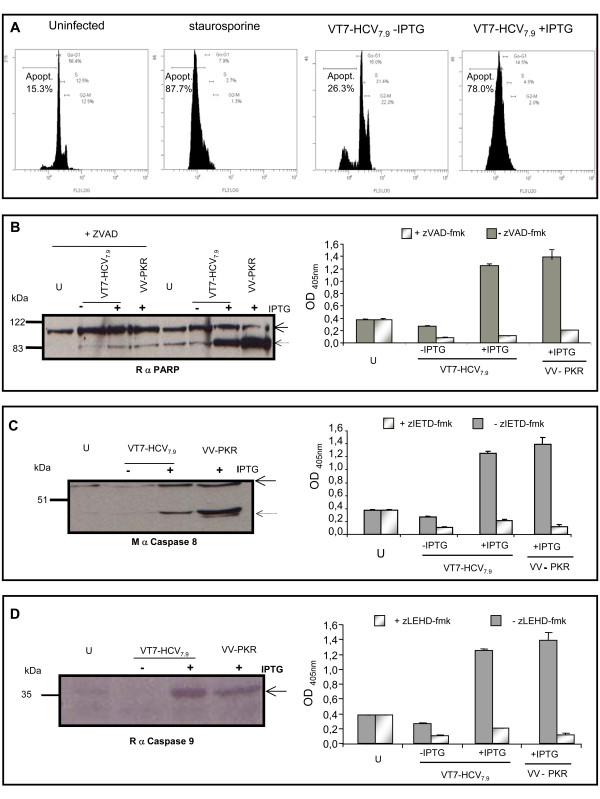
**HCV proteins induced apoptosis in a caspase-dependent manner. A: **Extent of apoptosis. HeLa cells were infected at 5 PFU/cell with the recombinant VT7-HCV7.9 in the presence or absence of IPTG. At 24 h p.i, uninfected and infected cells where fixed with an EtOH 70%-PBS solution, washed and stained with propidium iodide (PI) as explained in Material and Methods. The cell cycle was measure by flow cytometry. Cells treated with staurosporine at 0.5 μM for 16 h were used as positive control. **B**: Activation of effector caspases. HeLa cells were infected at 5 PFU/cell with the recombinant VT7-HCV7.9 in the presence (+) or absence (-) of IPTG individually or in combination with a general caspase inhibitor, zVAD-fmk at 50 μM. Cell lysates from uninfected and infected cells were collected at 48 h p.i and separated on a 12% SDS-PAGE for immunoblot analysis using an antibody that recognizes full-length (116 kDa) and cleavage-PARP (89 kDa) (left panel) or used for the quantification of the apoptotic levels by ELISA **(right panel)**. **C**: Caspase-8 activation. HeLa cells were infected at 5 PFU/cell with the recombinant VT7-HCV7.9 individually or in combination with a caspase-8 inhibitor, zIEDT-fmk at 50 μM, in the presence (+) or absence (-) of IPTG. Uninfected and infected cell lysates were collected at 48 h p.i. and separated on a 12% SDS-PAGE for immunoblot analysis using an antibody that recognizes procaspase- (57 kDa) and active-caspase-8 (43 kDa) (left panel) or used for the quantification of the apoptotic levels by ELISA (right panel). **D**: Caspase-9 activation. HeLa cells were infected at 5 PFU/cell with the recombinant VT7-HCV7.9 individually in the presence (+) or absence (-) of IPTG or in combination with a caspase-9 inhibitor, zLEHD-fmk at 50 μM. Uninfected and infected cell lysates were collected at 48 h p.i and separated on a 12% SDS-PAGE for immunoblot analysis using an antibody that recognizes the active-caspase 9 (37 kDa) (left panel) or used for the quantification of the apoptotic levels by ELISA (right panel). Cells infected with the inducible VV-PKR were used as positive control.

To quantify the extent of apoptosis, DNA content was analyzed by flow cytometry after permeabilization and labelling with the DNA-specific fluorochrome propidium iodide. As shown by flow cytometry, 78% of HeLa cells infected with VT7-HCV_7.9 _in the presence of IPTG were apoptotic in contrast with the 26% determined when cells were infected with VT7-HCV_7.9 _in the absence of the inducer (Fig. 6A).

Since DNA fragmentation represents a late apoptotic event, we analyzed the activation of effector caspases as a previous stage in the induction of apoptosis. Apoptotic caspases are activated by a proteolytic cascade resulting in the cleavage of critical cellular substrates, including lamins and poly (ADP-ribose) polymerase (PARP). By immunoblot analysis using anti-poly-(ADP-ribose) polymerase (PARP) antibody, which recognizes the full (116 kDa) and cleaved (89 kDa) form of PARP, we determined that the expression of HCV proteins induced the activation of effector caspases, as revealed by the presence of the 89 kDa cleaved form in cell extracts from VT7-HCV_7.9 _infected cells in the presence of IPTG. This activation was similar to that obtained in cells expressing the apoptotic inducer protein kinase PKR used as positive control. In contrast, minimal PARP cleavage product was observed in cell extracts from both uninfected cells and VT7-HCV_7.9 _infected cells in the absence of IPTG (Fig. 6B, left panel). The general caspase inhibitor zVAD-fmk blocked completely activation of caspases, as revealed by PARP cleavage and by ELISA (Fig. 6B).

Having established the activation of downstream effector caspases by HCV polyprotein expression, we set out to analyze the upstream or initiator caspases that exert regulatory roles, like caspase-8 and 9. Western blot analysis of lysates from VT7-HCV_7.9 _infected cells in the presence of IPTG using an antibody that recognizes the active caspase-8, detected a cleaved product of 43 kDa, which corresponds to the active subunit of caspase-8, and a product of 57 kDa, which corresponds to pro-caspase-8 (Fig. 6C, left panel). The same size cleaved product was also observed in cell lysates from VV-PKR infected cells used as positive control. In contrast, in uninfected cells or in cells infected with VT7-HCV_7.9 _in the absence of IPTG only the pro-caspase-8 product was observed. Caspase-8 activation and apoptosis induction in cells infected with VT7-HCV_7.9 _in the presence of IPTG was strongly inhibited by pre-treating the cells with the specific caspase-8 inhibitor zIETD-fmk (Fig. 6C, right panel). These results showed that expression of HCV proteins induces caspase-8-mediated apoptosis.

To define if the mitochondrial route could also be involved in the apoptosis induced by HCV polyprotein expression, we analyzed by Western blot the activation of caspase-9. Lysates from uninfected and VT7-HCV_7.9_infected cells in the presence or absence of IPTG were reacted with a specific antibody that detects only the cleaved product of 37 kDa corresponding to the active subunit of caspase-9 (Fig. 6D). The active caspase-9 was detected in lysates from cells expressing the HCV proteins in contrast to lysates from uninfected cells or from cells infected with VT7-HCV_7.9 _in the absence of IPTG (Fig. 6D, left panel). The activation of caspase-9 was confirmed after quantification by ELISA of the extent of the apoptosis induced by HCV proteins in the presence or absence of the specific caspase-9 inhibitor zLEHD-fmk. Severe inhibition was obtained by pretreating cells infected with VT7-HCV_7.9 _in the presence of IPTG with zLEHD-fmk (Fig. 6D, right panel). The above observations establish that HCV proteins activate initiator and effector caspase-dependent death processes, with involvement of the two caspase 8 and 9 pathways.

### Identification of differentially expressed human genes in cells expressing HCV proteins

Since identification of host genes triggered in response to HCV proteins is important to understand the pathogenic effects of HCV virus, we performed a microarray analysis to profile transcripts differentially expressed in HeLa cells infected with VT7-HCV_7.9 _that inducible express HCV proteins. A comparative analysis of genes regulated was done after subtracting the values obtained from cells infected in the absence of the inducer IPTG versus values obtained in the presence of IPTG. Hybridization analysis revealed that at 6 hours after induction of HCV polyprotein expression 231 genes were differentially regulated. About 71% of these genes appear upregulated whereas the remaining 29% were downregulated. At 16 h post-induction the number of transcripts that passed the filtering conditions is significantly reduced. 81 genes were differentially expressed at this time and only 43% of them appear upregulated (see Additional file 1). The reduction observed at 16 h p.i correlated with the cell damage induced by HCV proteins in the infected cells, and hence only the data from 6 h p.i will be considered. Real time RT-PCR was used to verify the transcriptional change in selected genes, as detected by microarray (Table 1) since we have previously verified that microarray data correlated well with RT-PCR quantification [22,23].

**Table 1:** Confirmation of microarray data by real time RT-PCR

**Gene product**	**Fold change by:**			
	**Microarray**		**RT-PCR**	
	**t = 6**	**t = 16**	**t = 6**	**t = 16**

**H3F3B**	2.65	1.39	2.07	2.28
**HIST2H4A**	3.67	8.45	3.82	8.65
**IL6**	5.67	7.49	8.16	10.1

Most of the biochemical and morphological changes induced by HCV proteins described in this study were reflected in the gene expression profiling. Genes involved in apoptosis, oxidative stress, mitochondrial functions or membrane transport were upregulated by HCV proteins (Table 2). Moreover, genes encoding proteins implicated in lipid metabolism, DNA binding, cell cycle, signalling and inflammatory response changed in expression throughout the infection.

**Table 2:** Microarray analysis revealed characteristic changes in gene expression profiling of HeLa cells during HCV protein expression from VT7-HCV_7.9 _(6 h p.i)

**GENE DESCRIPTION**	**GENBANK ACCESSION**	**GENE SYMBOL**	**FOLD-CHANGE**
**Apoptosis**			
RAD21 homolog (S. pombe)	NM_006265	RAD21	**2,93**
Protein phosphatase 2 (formerly 2A), catalytic subunit, alpha isoform	NM_002715	PPP2CA	**2,07**
Hepatocellular carcinoma-associated antigen 66	NM_018428	HCA66	**1,7**
Glucose regulated protein, 58 kD	NM_005313	PDIA3	**1,67**
Insulin-like growth factor 1 receptor	NM_000875	IGF1R	**-1,64**
Sphingosine kinase type 2 isoform	BC006161	SPHK2	**-1,87**
			
**Mitochondrial functions**			
ATP synthase, H+ transporting, mitochondrial F1 complex	NM_005174	ATP5C1	**2,6**
ATP synthase, H+ transporting, mitochondrial F1 complex, O subunit	NM_001697	ATP5O	**2,48**
Complement component 1, q subcomponent binding protein	NM_001212	C1QBP	**2,27**
NADH dehydrogenase (ubiquinone) 1 beta subcomplex, 9 (22 kD, B22)	NM_005005	NDUFB9	**2,21**
Voltage-dependent anion channel 1	NM_003374	VDAC1	**1,86**
Surfeit 1	NM_003172	SURF1	**1,69**
Solute carrier family 25, member 10	NM_012140	SLC25A10	**-2,41**
			
**Lipid metabolism/Oxidative stress**			
DnaJ (Hsp40) homolog, subfamily C, member 10	AK027647	DNAJC10	**3,68**
Glutathione peroxidase 4 (phospholipid hydroperoxidase)	NM_002085	GPX4	**2,05**
Fatty acid binding protein 5 (psoriasis-associated)	NM_001444	FABP5	**1,81**
Nuclear receptor subfamily 5, group A, member 2	NM_003822	NR5A2	**1,81**
Peroxiredoxin 1	NM_002574	PRDX1	**1,71**
StAR-related lipid transfer (START) domain containing 4	AK054566	STARD4	**1,63**
Cytochrome P450, family 19, subfamily A, polypeptide 1	NM_031226	CYP19A1	**1,57**
Glutathione S-transferase M1	NM_000561	GSTM1	**-1,65**
ATPase, class I, type 8B, member 4	NM_024837	ATP8B4	**-1,58**
24-dehydrocholesterol reductase	NM_014762	DHCR24	**-1,81**
Peripheral myelin protein 2	NM_002677	PMP2	**-1,84**
Glucose-6-phosphate dehydrogenase	NM_000402	G6PD	**-2,38**
			
**Membrane transport**			
Clathrin, light polypeptide (Lca)	NM_007096	CLTA	**3,05**
Centaurin, gamma 2	NM_014914	CENTG2	**2,1**
Adaptor-related protein complex 3, sigma 1 subunit	NM_001284	AP3S1	**1,76**
Coatomer protein complex, subunit beta	NM_016451	COPB1	**1,73**
USO1 homolog, vesicle docking protein (yeast)	NM_003715	USO1	**1,73**
SEC24 related gene family, member B (S. cerevisiae)	NM_006323	SEC24B	**1,72**
Paralemmin	NM_002579	PALM	**1,67**
Adaptor-related protein complex 2, mu 1 subunit	NM_004068	AP2M1	**-1,85**
Lectin, mannose-binding 2-like	NM_030805	LMAN2L	**-1,89**
Reticulon 4	AF148537	RTN4	**1,64**
			
**DNAbinding/Cell cycle**			
Histone cluster 1, H2am	NM_003514	HIST1H2AM	**7,03**
Histone cluster 1, H4h	NM_003543	HIST1H4H	**6,09**
Histone cluster 2, H4a	NM_003548	HIST2H4A	**3,67**
H2A histone family, member Z	NM_002106	H2AFZ	**3,58**
Histone cluster 1, H4d	NM_003539	HIST1H4D	**2,68**
H3 histone, family 3B (H3.3B)	AF218029	H3F3B	**2,65**
CDC28 protein kinase regulatory subunit 2	NM_001827	CKS2	**2,21**
Karyopherin alpha 2 (RAG cohort 1, importin alpha 1)	NM_002266	KPNA2	**1,78**
Nuclear receptor subfamily 5, group A, member 2	NM_003822	NR5A2	**1,81**
			
**Inflammatory response/Signalling**			
Interleukin 6 (interferon, beta 2)	NM_000600	IL6	**5,67**
Chloride intracellular channel 1	NM_001288	CLIC1	**2,81**
Neuroepithelial cell transforming gene 1	BC010285	NET1	**2,28**
Interleukin-1 receptor-associated kinase 1	NM_001569	IRAK1	**2,23**
CDC42 small effector 1	NM_020239	CDC42SE1	**1,73**
			
**Others**			
DEAD (Asp-Glu-Ala-Asp) box polypeptide 5	NM_004396	DDX5	**2,91**
TP53RK binding protein	NM_016058	TPRKB	**2,51**
T-complex 1	NM_030752	TCP1	**2,43**
Eukaryotic translation initiation factor 4E	NM_001968	EIF4E	**2,23**
Olfactomedin 1	NM_014279	OLFM1	**2,03**
Leucine zipper, down-regulated in cancer 1	NM_012317	LDOC1	**-1,66**
HtrA serine peptidase 3	AY040094	HTRA3	**-1,84**

Genes implicated in apoptosis such as RAD21, PPP2CA, HCA66 or PDIA3 were upregulated whereas antiapoptotic genes IGF1R or SPHK2 were downregulated by HCV proteins. Interestingly, it has been described that HCA66 is able to modulate selectively Apaf-1 dependent apoptosis increasing downstream caspase activity following cytochrome c release from the mitochondria [24], an event observed during the inducible expression of HCV proteins in our virus-cell system. Within the group of genes related with mitochondrial functions, the C1QBP and SLC25A10 transcripts have been correlated with HCV infection. C1QBP gene appears upregulated in liver biopsies from acutely HCV-infected chimpanzees whereas downregulation of SLC25A10 alters mitochondrial and cellular status resulting in altered susceptibility of hepatic cells to apoptosis [25].

HCV proteins also induced disturbance in the expression of lipid metabolism and oxidative stress. Upregulation of GPX4, PRDX1 and CYP19A1 genes have been previously detected in biopsies of HCV infected chimpanzees or in human hepatocellular carcinoma (HCC) samples [25-27]. In contrast, it was reported that GSTM1 null genotype may facilitate HCV infection becoming chronic [28], and also this gene was downregulated in liver cells expressing entire HCV ORF [29]. Glucose-6-phosphate dehydrogenase (G6PD) activity was inhibited in hyperplastic liver as well as in HCC [30].

In agreement with the alterations and formation of electron dense structures observed in infected cells expressing the HCV polyprotein, genes such as CLTA, CENTG2 or AP3S1, which are closely related with the membrane dynamics, were upregulated. Moreover, ER-resident proteins like DNAJC10 and Reticulon 4 (RTN4), which modulate the ER morphology under stress conditions, also appear activated in HCC samples [31,32]. Gene encoding DNA binding proteins such as HIST1HA2M, HIST1H4H, HIST2H4A, H2AFZ and HIST1H4D, or cell cycle transcripts (CKS2 or KPNA2), were consistently upregulated. Specific increases in histones and cyclin genes were markers of proliferative changes detected in the liver of HCV infected chimpanzees [25,33].

Other genes that have been associated with HCV infection and were differentially expressed in our system are CLIC1, NET1, IRAK1, DDX5, TPRKB, TCP1, OLFM1, LDOC1 and HTRA3. CLIC1 gene was upregulated in liver biopsies from infected chimpanzees [25], whereas DDX5 helicase has homology with DDX3, which plays an important role in HCV replication [34]. Relative high levels of NET1 and IRAK1 were reported in HCC [35,36]. Genes encoding for proteins TPRKB, T-Complex 1 (TCP1), Olftactomedin 1 (OLFM1), LDOC1 and HTRA3 have been implicated positive or negatively in cancer progression. TPRKB protein acts as a potential inhibitor of the binding of p53-related protein kinase PRPK to p53 [37], whereas T-Complex 1 and Olftactomedin 1 promote proliferation of cancer cells [38,39]. On the other hand, it has been suggested that the downregulation of LDOC1 and HTRA3 genes may play an important role in the development and/or progression of some cancers [40,41].

Overall, the association of the gene expression profile obtained after induction of HCV proteins in VT7-HCV_7.9 _infected HeLa cells with genomic changes in HCV pathogenesis highlights the biological significance of the morphological and biochemical events identified in this study.

## Discussion

Various *in vitro *model systems have been developed to study the role of HCV polyprotein expression on host cell responses [4,6,42-45]. However, only recently was described a system that allows the growth of HCV in cultured cells [7,9]. Although these systems produced infectious HCV, the virus yields are low, not all cells become infected and the virus growth is only observed in certain cell lines. In this study we used the poxvirus-based system because it allowed the regulated expression of the nearly entire HCV polyprotein (except the C-terminal part of NS5B) in a wide range of cell types that efficiently support the VACV infection [46]. Confocal (CM) and electron microscopy (EM) were used to determine the subcellular localization of HCV proteins and the intracellular changes that occurred during the course of infection.

Comparable to previous analysis of HCV proteins expressed in culture cells [47,48], the HCV polyprotein expressed from the VT7-HCV_7.9 _recombinant virus in the presence of the inducer IPTG, was localized largely in the cytoplasm, with a reticular/punctuate distribution that was more intense in the perinuclear area. In the course of infection there is disruption of the Golgi apparatus and co-localization between ER markers and HCV proteins. Partial co-localization between HCV and mitochondrial proteins was also detected. EM analysis showed the induction of membrane alterations similar to those found by other groups in cell-culture systems [15,48] or in human and primate liver biopsies [49-51]. The main structures observed in infected HeLa and hepatic HepG2 cells were the formation of cytoplasmic "membrane webs", similar to those observed by Egger et al. [15]. These appear as electron dense structures (EDS) dispersed in several areas of the cell cytoplasm. As revealed by immunofluorescence, EM and immunoelectron microscopy (IEM) there is a clear loss of ER organization and concentration of the gold particles around the membranous webs. The electron dense structures were coated with an outer membrane connected to the ER membrane, where it has been described that HCV envelope proteins (E1 and E2) and nonstructural proteins are localized [48,52,53]. In infected cells expressing the HCV polyprotein we detected by EM the emergence of some "virus-like particles" structures. The shape of these structures seemed typical of mature virions of flavivirus [54]. Their size of 40 nm are similar to the virion-like structures observed in HeLa cells transfected with the full-length sequence of the HCV genome [6], but slightly smaller than the 55-nm virus-like particles recovered from the circulation on an HCV-infected host [55]. Nonetheless, they are consistent with the size estimated for chimpanzee infectivity in a filtration study [56] and the size of a tissue culture-derived virus like particle [57]. We failed to detect HCV particles with enclosed envelopes corresponding to the full viral particles, probably because of removal of the 5' and 3' terminal regulatory regions of HCV genome in VT7-HCV_7.9, _the lack of an entire NS5B protein and/or because the process of envelope acquisition is slow or transient and affected by specific cellular host protein(s) [58].

NS4B and NS5A expressed from the near full-length HCV genome produced strong labelling concentrated in the cytoplasm and were associated with the membranous webs. While the significance of the observed membrane alterations induced by HCV proteins cannot be assessed, it has been recently proposed that HCV genome synthesis occurs at lipid droplets-associated sites attached to the ER in virus-infected cells [59,60] and that HCV assembly and maturation occurs in the ER and post-ER compartments [61]. Hence, the observations that NS4B and NS5A proteins are associated with the membranous web and that the same structure is found during HCV replication in chimpanzee liver, make the membranous web, a good candidate to act as the replication complex. In agreement with previous observations [61-63], our results provide evidence that the Golgi complex and the ER are subcellular compartments directly involved in HCV morphogenesis.

Other cellular alteration observed by EM in HeLa and HepG2 cells expressing the HCV polyprotein was the presence of swelling mitochondria, a phenomenon that has been previously described in patients with chronic HCV [64]. Since partial co-localization between HCV proteins and mitochondrial markers was also detected by immunofluorescence in our VACV system, here we characterized biochemically to what extent HCV polyprotein expression alter mitochondrial homeostasis. We observed by CM that in HeLa cells infected with VT7-HCV_7.9 _in the presence of the inducer IPTG there is release of cytochrome c from the mitochondria. This release correlates with the disruption of the mitochondrial membrane potential, as revealed by the high proportion of cells with decreased ΔΨm, and by the high levels of ROS. It has been reported that some HCV proteins, in addition to the ER, localize in the mitochondria disturbing its function. The structural core protein targets the mitochondria and increases Ca^2+ ^dependent ROS production [65,66]. NS4A, when inducibly expressed in HepG2 transfected cells, is located in the mitochondria and is implicated in the loss of ΔΨm [67], while when expressed from an HCV RNA replicon it forms a complex with NS3 changing the intracellular distribution of this organelle, triggering mitochondrial damage as evidence by the collapsed ΔΨm and by the release of cytochrome c into the cytoplasm [13]. Although we can not assign the mitochondrial disturbance function to any HCV protein expressed in our system, it seems clear the need for the combined action of some HCV proteins. Our results are compatible with those obtained in cell lines expressing the entire HCV ORF where a profound effect on cell oxidative metabolism, depression of mitochondrial membrane potential and increased production of ROS were reported [68]. Functional analysis of human liver biopsies suggest the impairment of key mitochondrial processes, as those described above, during advance stages of fibrosis, evidencing the association between oxidative stress and hepatic mitochondrial dysfunction with HCV pathogenesis [69].

Several *in vitro *studies revealed that synthesis of HCV structural proteins or the full-length genome have a direct cytotoxic effect or activate an apoptotic response [13,21,70,71]. Furthermore, the alteration of ER membranes [15] and the activation of signalling pathways characteristic of an ER-stress condition, have been found to be associated with the expression of HCV proteins [72-74]. Although these data suggest that HCV may alter intracellular events with possible consequences on liver pathogenesis, the complex mechanism and the role of the viral proteins implicated is under extensive study. Here we showed that HCV polyprotein expression from a VACV recombinant triggered morphological features of apoptosis, such as membrane blebbing and cell shrinkage, that have been described as indicative of cytoskeleton rearrangement due to apoptosis [75,76]. Nuclear DNA fragmentation was also observed, as previously examined by others groups using TUNEL staining assay with serum from HCV infected patients [77]. As DNA fragmentation represents a late apoptotic event, we investigated the activation of caspases which are documented to play an important role in the apoptosis detected in various liver disease [78,79]. Moreover, the importance of caspases in hepatitis is underscored by studies with pharmacological caspase inhibitors, which potently suppressed experimental hepatitis [80,81].

We found that expression of HCV proteins from the VT7-HCV_7.9 _recombinant increased the activity of initiator and effector caspases and induced apoptosis in a caspase-dependent manner; these effects were completely prevented by treatment with specific caspase inhibitors. This activation has been previously observed in cell culture systems individually expressing Core or E2 structural proteins [71,82] and in the HCV RNA replicon when all HCV proteins are produced [13].

The subcellular forms and biochemical effects triggered by HCV proteins had a profound effect on gene profiling as determined by microarrays. We found up and down regulation in the transcription pattern of several genes associated with lipid metabolism, oxidative stress, apoptosis, mitochondrial dysfunction and cellular proliferation. Since modulation of these genes has been associated with HCV pathogenesis, it suggest that the VAC system expressing the HCV polyprotein impact the host cell somewhat similar as during HCV infection. Thus, the VACV based system is a valuable model in which to investigate critical features of HCV infection and morphogenesis, to characterize virus-host cell interactions and to test the effect of antiviral drugs in the different cell injuries associated with liver diseases.

## Methods

### Cells and viruses

Cells were maintained in a humidified air 5% CO_2 _atmosphere at 37°C. Human HeLa and monkey BSC40 cells were grown in Dulbecco's modified Eagle's medium (DMEM) supplemented with 10% newborn calf serum (NCS). Human HepG2 hepatocellular carcinoma cells (ATCC HB-8065) were maintained in DMEM supplemented with 10% fetal calf serum (FCS).

The recombinant VT7-HCV_7.9_, derived from the vaccinia Western Reserve strain (VACV-WR), has been previously described [12]. It contains 7.9 Kb of the HCV ORF from genotype 1b inserted within the viral HA locus under the transcriptional control of the T7 promoter, and expresses the T7 RNA polymerase upon induction with IPTG. The recombinant VV-PKR expressing IPTG-inducible dsRNA-dependent protein kinase (PKR) was generated by homologous recombination of their respective pPR35-derived plasmid with the VACV-WR strain as previously described [83]. Viruses were grown and titrated in BSC40 cells and purified by banding on sucrose gradients [84].

### Immunofluorescence

HeLa cells cultured on coverslips were infected at 5 PFU/cell with VT7-HCV_7.9 _in the presence or absence of IPTG (1.5 mM final concentration). At 24 h p.i, cells were washed with PBS, fixed with 4% paraformaldehyde and permeabilized with 2% Triton X-100 in PBS (room temperature, 5 min). To detect the mitochondria, cells were stained *in vivo *with Mitotracker Deep Red 633 (Molecular Probes) at 500 nM in DMEM, before fixing the cells. After blockade, cells were incubated for 1 h at 37°C with the specific primary antibodies. The coverslips were then extensively washed with PBS, followed by incubation in the dark for 1 h at 37°C with specific secondary antibodies conjugated with Alexa 488 (green), Alexa 594 (red) or with the green fluorochrome Cy2 (purchased from Molecular Probes), and with the DNA staining reagent ToPro-3 (diluted 1:200). Images were obtained by the Bio-Rad Radiance 2100 confocal laser microscope at a resolution of 63X, collected by Lasersharp 2000 software and processed in LaserPix.

### Electron microscopy

#### Embedding of infected cells in EML-812

Monolayers of HeLa or HepG2 cells were infected with 5 PFU/cell of VT7-HCV_7.9 _in the presence or absence of IPTG. After 16 h, cells were fixed in situ with a mixture of 2% glutaraldehyde and 1% tannic acid in 0.4 M HEPES buffer (pH 7.2) for 1 h at room temperature. Fixed monolayers were removed from the culture dishes in the fixative and transferred to Eppendorf tubes. After centrifugation and a wash with HEPES buffer, the cells were stored at 4°C until use. For ultrastructure studies, fixed cells were processed for embedding in the epoxy resin EML-812 (TAAB Laboratories, Ltd., Berkshire, UK) as previously described [85]. Postfixation of cells was done with a mixture if 1% osmium tetroxide and 0.8% potassium ferricyanide in distilled water for 1 h at 4°C. After four washes with HEPES buffer, samples were treated with 2% uranyl acetate, washed again, and dehydrated in increasing concentrations of acetone (50, 70, 90, and 100%) for 15 min each time at 4°C. Infiltration in resin was done at room temperature for 1 day. Polymerization of infiltrated samples was done at 60°C for 3 days. Ultrathin sections (40 to 60 nm thick) of the samples were stained with saturated uranyl acetate and lead citrate by standard procedures. Collections of images were done in a JEOL 1200-EX II electron microscope operating at 100 kV.

#### Embedding of infected cells in Lowicryl K4M

Monolayers of HeLa cells were infected with 5 PFU/cell of VT7-HCV_7.9 _in the presence or absence of IPTG. After 16 h, cells were fixed in situ with a mixture of 4% paraformaldehyde and 0.1% glutaraldehyde in PBS for 30 minutes at 4°C. Fixed cells were then removed from the dishes and processed for low-temperature embedding in Lowicryl K4M. After extensive washing with PBS, the cells were incubated for 20 minutes with a solution of 0.2 M ammonium chloride, to block any possible free aldehyde groups that may remain in the preparations. Small pellets of chemically fixed cells were cryoprotected with glycerol and quick frozen in liquid propane. Frozen specimens were processed by freeze-substitution for 48 h at -90°C in a mixture of methanol and 0.5% (wt/vol) uranyl acetate. Samples were then treated at -30°C with a mixture of Lowicryl K4M:methanol (1:3) for 1 hour, Lowicryl K4M:methanol (1:1) for 1 hour, Lowicryl K4M:methanol (3:1) for 1 hour, followed by an overnight incubation in 100% Lowicryl. After replacing the resin with a fresh one, samples were kept at -30°C for 8 hours. Finally, the samples were transferred to capsules and polymerized with ultraviolet light for one day at -30°C, and two days at room temperature.

#### Immunogold labeling of ultrathin sections

Immunogold localization on sections of infected cells was performed by placing the sections on drops of different solutions. After a 30 min incubation with Tris-HCl buffer gelatine (TBG) (30 mM Tris-HCl, pH 8.0, containing 150 mM NaCl, 0.1% BSA, and 1% gelatin) to block non-specific binding of the antibodies to the samples, sections were floated for 60 min on a drop of the specific primary antiserum, diluted in TBG. After jet-washing with PBS, grids were floated on 4 drops of TBG and incubated 10 min on the last drop before a 45 min incubation with the secondary antibody, a goat anti-rabbit immunoglobulin G conjugated with colloidal gold of 10 nm, or goat anti-mouse IgG+igM conjugated with colloidal gold of 5 or 10 nm that was purchased from BioCell (Cardiff, UK). Washing was repeated as before, and grids were then floated on several drops of distilled water before staining with a solution of saturated uranyl acetate for 20 min. For double-labelling experiments, representative signals corresponding to both primary antibodies were obtained after testing different combinations of labelling steps.

#### Imaging and measurements

Regular thin sections were collected on formvar-coated gold grids of 200 meshes, stained, and studied by EM. Ultrathin sections of the samples were either stained by standard procedures, stained with saturated uranyl acetate in 70% ethanol (procedure that improves contrast), or processed for immunogold labelling. Collection of images and measurements were done with a JEOL 1200-EX II electron microscope operating at 100 kV.

#### Quantification of mitochondrial membrane potential (ΔΨm) and production of reactive oxygen species (ROS)

Mitochondrial membrane potential was quantified by flow cytometry. Infected and uninfected floating and adhered HeLa cells were collected at 48 h p.i from the wells, centrifuged at 2500 rpm for 15 min at 25°C, washed once with PBS and resuspended in 1 ml of PBS containing 0.2 μM TMRE during 30 min at 37°C, in the dark. TMRE fluorescence was acquired through the FL-2 channel (575 nm). Bivariate flow cytometry using a FACScan was performed acquiring 10000 events per sample with fluorescence signals at logarithmic gain analysed with EXPO32 analysis software. The production of reactive oxygen species (ROS) was monitored at 48 h p.i by staining cells with 2-HE and analysed by FACScan. Cells were treated as indicated above, harvested, and washed with PBS. The pellet was resuspended in MIB buffer [86] and incubated with 2 μM of 2-HE for 30 min at 37°C in the dark. Analysis was carried out by flow cytometry; 2-HE was measured in FL2 as described above. In both assays staurosporine treated cells were used as positive control.

### Measurement of apoptotic cell death

#### By cell cycle analysis

The different stages of cell cycle and the percentage of cells with subG_0_DNA content were analyzed by propidium iodide (PI) staining as previously described [87]. HeLa cells were infected at 5 PFU/cell with VT7-HCV_7.9_, in the presence or absence of the inducer IPTG. At 24 h p.i uninfected and infected cells were removed by pipetting, washed once with cold PBS, and permeabilized with 70% ethanol in PBS at 4°C overnight. After three washes with PBS, the cells were incubated for 45 min at 37°C with RNAse-A (0.1 mg/ml) and stained with PI (10 μg/ml) during 15 min at room temperature. The percentage of cells with hypodiploid DNA content was determined by flow cytometry acquiring 15000 events per sample. Cells treated with 0.5 μM of staurosporine (Sigma) for 16 h were used as a positive control of apoptosis induction.

### By ELISA

HeLa cells were infected as described above in the presence or absence of general and specific caspase inhibitors and harvested at 24 h p.i. The extent of apoptosis was determined using the cell death detection enzyme-linked immunosorbent assay (ELISA) kit (Roche) according to the manufacturer's instructions. Duplicate samples were measured in two independent experiments. Cells infected with VV-PKR in presence of IPTG were used as positive control. The specific inhibitors of caspase 8 (zIETD-fmk), caspase 9 (zLEHD-fmk) and the general caspases inhibitor (zVAD-fmk) were added to the cells after one hour of virus adsorption at a final concentration of 50 μM (Calbiochem).

#### Analysis by Western blot of active caspases

To examine expression of active caspases-8 and 9, HeLa cell monolayers were infected with 5 PFU/cell of VT7-HCV_7.9_, in the presence or absence of the inducer IPTG. Uninfected and infected cells were collected at 48 h p.i. in lysis buffer (50 mM Tris-HCl pH 8.0, 0.5 M NaCl, 10% NP40, 1% SDS). Equal amounts of protein lysates were separated by 12% SDS-PAGE, transferred to nitrocellulose membranes and reacted with a primary rabbit antibody against cleaved caspase-9 or with a primary mouse antibody against cleaved caspase-8, followed by the respective secondary antibody. The activation of effector caspases was similarly assayed using a primary rabbit anti-poly-(ADP-ribose) polymerase (PARP) antibody, which recognizes the full (116 kDa) and cleaved (89 kDa) form of PARP.

#### Microarray analysis

Total RNA was isolated from HeLa cells infected at 5 PFU/cell with VT7-HCV_7.9 _in the presence or absence of IPTG at 6 and 16 h p.i with Ultraspect_II RNA (Biotecx, Houston, TX), following manufacturer's instructions. RNA was purified with Megaclear (Ambion, Foster City, CA), and the integrity was confirmed by using an Agilent (Santa Clara, CA) 2100 Bioanalyzer. Two independent replicates were processed for analysis. Total RNA (1.5 μg) was amplified with an Amino Allyl MessageAmp aRNA kit (Ambion); 54 to 88 μg of amplified RNA (aRNA) was obtained. The mean RNA size was 1,500 nucleotides, as observed using the Agilent 2100 Bioanalyzer. For each sample, 6 μg aRNA was labeled with one aliquot of Cy3 or Cy5 Mono NHS Ester (CyDye postlabeling reactive dye pack; GE Healthcare) and purified using Megaclear. Incorporation of Cy5 and Cy3 was measured using 1 μl of probe in a Nanodrop spectrophotometer (Nanodrop Technologies). For each hybridization, Cy5 and Cy3 probes (150 mol each) were mixed and dried by speed vacuum and resuspended in 9 μl RNase-free water. Labeled aRNA was fragmented by adding 1 μl 10× fragmentation buffer (Ambion), followed by incubation (70°C for 15 min). The reaction was terminated with the addition of 1 μl stop solution (Ambion) to the mixture. Two dye-swapped hybridizations were performed for each comparison; in one, the induced-infected sample was Cy3 labeled, and the non-induced-infected sample was Cy5 labeled; in the second, labeling was reversed. Double labeling was used to abolish dye-specific labeling and hybridization differences.

#### Slide treatment and hybridization

Slides containing 22,264 spots (21329 different oligonucleotides) corresponding to Human Genome Oligo set version 2.2 (QIAGEN, Hilden, Germany) were obtained from the Genomic and Microarrays Laboratory (Cincinnati University, Cincinnati, OH). Information about printing and the oligonucleotide set can be found on their website . Slides were prehybridized and hybridized as described previously [23]. Images from Cy3 and Cy5 channels were equilibrated and captured with an Axon 4000B scanner, and spots were quantified using GenePix 5.1 software. Data for replicates were analyzed using Almazen software (Bioalma, Spain). Briefly, background was subtracted from the signal, Log10 (signal) was plotted versus Log2 (ratio) and Lowess normalization used to adjust most spots to Log Ratio 0. This was calculated for all four replicates and a table was obtained with mean signal, x-fold change, Log Ratio, standard deviation of the Log Ratio, z-score and p-value [88]. Log Ratio and x-fold change were obtained by substracting the non-induced-infected sample gene expression values from those obtained in the induced-infected samples. In each analysis, genes with an interreplicate mean signal of < 50 or a p-value > 0.1 were filtered out.

#### Quantitative real-time RT-PCR

RNA (1 μg) was reverse-transcribed (RT) using the superscript first-strand synthesis system for reverse transcription-PCR (RT-PCR) (Invitrogen). A 1:40 dilution of the RT reaction mixture was used for quantitative PCR. Primers and probe set used to amplify IL-6, H3F3B, and HIST2H4A were purchased from Applied Biosystems. RT-PCR reactions were performed according to Assay-on-Demand, optimized for TaqMan Universal PCR MasterMix, No AmpErase UNG, as described [22]. All samples were assayed in duplicate. Threshold cycle (Ct) values were used to plot a standard curve in which Ct decreased in linear proportion to the log of the template copy number. The correlation values of standard curves were always > 99%.

## Competing interests

The authors declare that they have no competing interests.

## Authors' contributions

AMV designed and performed the experiments and drafted the manuscript. CEG designed the study, analyzed the data and wrote the paper. CP carried out the electron microscopy studies. EDG performed the experiments. SG carried out the microarrays studies. JMG participated in the analysis of microarray data. ME conceived the study, and participated in its design, coordination and writing. All authors read and approved the final manuscript.
